# Mentalizing skills do not differentiate believers from non-believers, but credibility enhancing displays do

**DOI:** 10.1371/journal.pone.0182764

**Published:** 2017-08-23

**Authors:** David. L. R. Maij, Frenk van Harreveld, Will Gervais, Yann Schrag, Christine Mohr, Michiel van Elk

**Affiliations:** 1 University of Amsterdam, Department of Psychology, Amsterdam, The Netherlands; 2 University of Kentucky, Department of Psychology, Lexington, Kentucky, United States of America; 3 University of Lausanne, Institute of Psychology, Lausanne, Switzerland; Universite de Bretagne Occidentale, FRANCE

## Abstract

The ability to mentalize has been marked as an important cognitive mechanism enabling belief in supernatural agents. In five studies we cross-culturally investigated the relationship between mentalizing and belief in supernatural agents with large sample sizes (over 67,000 participants in total) and different operationalizations of mentalizing. The relative importance of mentalizing for endorsing supernatural beliefs was directly compared with credibility enhancing displays–the extent to which people observed credible religious acts during their upbringing. We also compared autistic with neurotypical adolescents. The empathy quotient and the autism-spectrum quotient were not predictive of belief in supernatural agents in all countries (i.e., The Netherlands, Switzerland and the United States), although we did observe a curvilinear effect in the United States. We further observed a strong influence of credibility enhancing displays on belief in supernatural agents. These findings highlight the importance of cultural learning for acquiring supernatural beliefs and ask for reconsiderations of the importance of mentalizing.

## Introduction

According to conservative estimates, at least 80% of the world population believes in intentional supernatural agents [1]. In this context, we refer to supernatural agents as an umbrella term for all intentional agents not conforming to a naturalistic worldview. Given this impressive number, the question arises what underlies this apparently universal human tendency to believe in intentional supernatural agents. One suggestion is that these beliefs emerge as by-products of normal evolved cognitive mechanisms, such as dualistic reasoning[2]. This suggestion is well established in the cognitive science of religion [3].

One of the key cognitive mechanisms hypothesized to underlie supernatural beliefs is the ability to mentalize or to engage in theory of mind (ToM) reasoning [2,4–17]. This is the ability to attribute intentions, beliefs, and desires to other minds [18,19]. The logic underlying this hypothesis is that in order for people to be able to believe in intentional supernatural agents, they should at least have the mentalizing abilities required to conceptualize the agent’s intentions [8,17]. Specifically, the idea is that an evolved cognitive mechanism for inferring intentionality of human agents is similarly activated when inferring the intentionality of supernatural agents. In the current study, we aimed to investigate whether mentalizing abilities are indeed important for supporting belief in supernatural agents, by investigating whether individual differences in mentalizing covary with degrees of belief. Also, we placed the relative importance of mentalizing in context by comparing it to the importance of credibility enhancing displays–the extent to which people observed credible religious acts during their upbringing [20–22].

In the existing literature, the relationship between mentalizing and belief in supernatural beliefs has been investigated in different ways. In one line of studies, researchers used the (shortened) Empathy Quotient [23,24], because mentalizing was argued to be important to empathy [16,17,25]. The link between the EQ and supernatural beliefs was found to be statistically significant, but modest (i.e., all *r*’s < .22). However, the EQ did not predict supernatural beliefs when variables such as analytic thinking or moral concern were taken into account [26]. Moreover, the psychometric validity of the scale has been critiqued, as the scale does not correlate to mentalizing ability tasks [27]. As a result, the EQ cannot be considered to unequivocally assess mentalizing. In other studies, taking into account a wider variety of operationalizations of mentalizing such as the reading the mind in the eye test and the perspective-taking task, the authors reported inconsistent relationships between mentalizing and supernatural beliefs [16,26]. The reading the mind in the eye test was significantly related to supernatural beliefs in the study of Norenzayan et al. [16] but not in the study of Jack et al. [26]. In sum, at most these studies demonstrated only a modest role for mentalizing underlying supernatural beliefs.

Another line of studies linking mentalizing with supernatural beliefs comes from studies focusing on people with autism spectrum disorder (ASD) or on neurotypical people's score at measures of ASD such as the Autism Spectrum Quotient [8,10,15,16,28–30]. People with ASD are thought to be characterized by difficulties conceptualizing intentions of others [18,31] and ASD seems to have a strong genetic component [32]. In two studies, researchers found people with ASD to have reduced supernatural beliefs compared to neurotypical people [16,33], but other researchers did not find such a relationship [34–36]. Moreover, anecdotal reports show that people with ASD can believe in supernatural agents [8,37–39] although they may endorse a more negative view of God [40]. In short, investigations into the relationship between ASD and supernatural beliefs have yielded mixed results.

In a final line of studies linking mentalizing to supernatural beliefs, researchers have linked brain areas associated with ToM (i.e., the so-called ToM-network) to supernatural beliefs and behaviors in neuroimaging studies [41]. The ToM-network is a network of functionally related brain regions that are steadily activated in association with tasks related to mentalizing [42], such as Heider and Simmel’s [43] classical Geometrical Figures Task (GFT). In this task, geometrical figures move as if they have intentions. The ToM-network encompasses the medial prefrontal cortex, the anterior and posterior cingulate cortex, the precuneus, and the bilateral temporal parietal junction [44–46]. In a study in which religious believers silently prayed to God, the ToM-network was found to be activated, whereas this was less the case when they thought of the Lord’s Prayer, made wishes to Santa Claus or thought of a nursery rhyme [47]. This finding suggests that personal contact with a supernatural agent involves ToM-related processing and this finding has been replicated and extended with a control condition in which participants imaginatively spoke to a loved one [48]. In a similar fashion, the ToM-network was activated when believers thought about God’s mental states [49] or God’s beliefs [50]. Finally, brain regions of the ToM-network were activated more strongly in supernatural believers than skeptics when randomly moving geometrical figures were shown [51]. Importantly, in this study the intensity of the activation in the ToM-network correlated with the intentionality ratings of the participants. Taken together, these neuroimaging studies seem to converge with the idea that naturally evolved brain mechanisms for ToM-reasoning are similarly activated when perceiving intentionality or when thinking about supernatural agents. Nevertheless, it is premature to conclude that mentalizing is an important cognitive mechanism enabling belief in supernatural agents, merely on the basis of these neuroimaging studies. Crucially, in these studies it is assumed that when the brain areas associated with the ToM-network are activated with a certain task this means that the underlying process (i.e., mentalizing) is active but this is not necessarily the case [36].

In short, the literature so far does not provide clear-cut evidence that mentalizing abilities are indeed a driving factor behind supernatural beliefs. Thus, to shed further light on this on-going debate, we extended earlier work in four important ways. First and foremost, in studies 2, 4 and 5, we compared the relative importance of mentalizing skills (as measured in the same way as previous researchers who observed effects of mentalizing) for predicting supernatural beliefs with a specific cultural learning theory on how supernatural beliefs are acquired [20–22]. According to some researchers, the role of culture in acquiring supernatural beliefs is secondary to primary intuitive cognitive biases [6]. Others acknowledge a strong reciprocal influence between cognitive biases and cultural factors [12,52]. However, there is a recent trend of researchers emphasizing the importance of cultural learning factors—they consider cognitive mechanisms to be secondary to cultural foundations of supernatural beliefs [20–22,53], with some even asking for a revision of the by-product framework [54]. Thus, to account for the current debate, we directly compared the relative importance of individual differences in mentalizing and exposure to credible religious displays during upbringing for predicting supernatural beliefs, and to our knowledge, we are the first to do so.

The theory of credibility enhancing displays (i.e., CREDs) is a cultural learning theory with a substantial explanatory potential. Henrich [20] and Lanman [21] have proposed that the extent to which people become supernatural believers is largely determined by the degree to which they have been exposed to credible displays of belief in the supernatural. For example, if parents or caretakers say they believe in God, pray every night before dinner and go to church every weekend, these are considered very credible displays of the existence of a supernatural realm. On the one hand, when CREDs of religiosity are observed, the likelihood is increased that observes take over supernatural beliefs expressed by actors. On the other hand, when CREDs of atheism or unreliable religious acts (e.g., highly unmoral religious actors) are observed, the likelihood is decreased that the observer acquires supernatural beliefs. Thus, CREDs provide a comprehensive explanation for both theism and atheism. Supportive data for the theory of CREDs have been presented [22,54], but a direct comparison with cognitive biases is missing.

A second way in which our study extends previous work on the relationship between mentalizing and belief is that large sample sizes were employed (Study 1–study 4) with over 67,000 participants in total. Therefore, we have strong foundations to draw conclusions from. A third way in which our study extends earlier work is that we made use of both self-report questionnaires (i.e., the EQ, AQ and hyper-systemizing, in order to directly compare our results with previous studies on this topic) as well as an experimental test used in neuroimaging studies to localize brain areas involved with ToM processing (i.e., the Geometrical Figures Task; in Study 4 and Study 5). Thereby, we increased the likelihood that we tapped into the concept of mentalizing more thoroughly than in most previous studies. Finally, we investigated samples from three different countries (i.e., The Netherlands [Study 1, Study 2 and Study 5], Switzerland [Study 3] and The United States of America [Study 4]) varying in the extent to which they are religious (i.e., secularized: With secularization we refer to the societal decline in level of religiosity), thereby improving the generalizability of our findings.

### Overview of the studies

As outlined above, we present five studies in which we cross-culturally investigated the relationship between mentalizing and supernatural beliefs in three countries varying in the extent to which they are secularized. We tested large samples, used different operationalizations of mentalizing and compared the relative importance of mentalizing to cultural learning (i.e., CREDs). We operationalized supernatural beliefs by several items indicative of religiosity (e.g., *‘To what extent do you belief in God*?*’*, ‘*To what extent do you consider yourself religious*?*’*), hence we refer to this concept as ‘religiosity’. In Study 1, we investigated the relationship between the AQ and religiosity in a large sample of participants from The Netherlands. In Study 2, we added the EQ and CREDs for a similar Dutch sample. In Study 3, we investigated the relationship between the AQ and religiosity in a less secularized country than The Netherlands (i.e., Switzerland). In Study 4, we investigated the relationship between the AQ, EQ, the geometrical figures task (as a more objective way of measuring mentalizing abilities) and compared the effects of these mechanisms in predicting religiosity to the role of CREDs in a pre-registered study (https://osf.io/6vrne/) with US participants. In Study 5 we compared adolescents from a Dutch high school specialized in ASD to adolescents from a regular high school. In all studies, we hypothesized a relationship between mentalizing abilities and religiosity, although we expected the relative influence of mentalizing abilities to be minimal compared to influences of cultural learning. Summing up, we investigated the relative contribution of mentalizing and CREDs on acquiring supernatural beliefs.

## Study 1: The Netherlands 1

### Materials and methods

#### Participants

In total, 99,516 participants started an online survey on the website of ‘Quest’, a popular Dutch Science magazine. Data were collected from the 8^th^ of April 2014 until the 14^th^ of January 2015. We excluded all participants who were younger than 18 years old (12,688 participants) and those who did not fill out the entire survey (21,267 participants). In total, 65,561 participants were used for further analyses. Participants (54.4% female) were on average 29.5 years old (*SD* = 11.1; range 18–85 years). All studies were approved by the ethical committee of the University of Amsterdam, confirmed to the laws applying to the countries in which they were conducted and were conducted in accordance with the declaration of Helsinki.

#### Procedure

On the website of Quest, participants were offered the opportunity to participate in an online survey (i.e., http://www.quest.nl/test/hoe-autistisch-ben-jij). The survey was also featured in an article on autism in the paper version of the magazine–offering participants the opportunity to get their personal score on the AQ. Before the survey started, participants were provided with some background information on autism. Participants were cautioned that the test was not an official diagnosis of autism, but rather an indication of their relative score on the autism spectrum in relation to the general population. For an official diagnosis, participants were referred to their general practitioner. The survey started with demographic questions, followed by the autism-spectrum quotient (AQ) questionnaire and subsequently participants received feedback about their scores. Participants were also given the option to fill out the shortened post-critical belief scale (Duriez, Soenens, & Hutsebaut, 2005), which was introduced by a short statement indicating that the researchers were interested in the relationship between autism and religious beliefs. The results of this questionnaire will be reported elsewhere.

#### Demographics

Participants were asked to report their gender, age and level of education (according to the Dutch educational system divided in 8 ordinal categories from no education to University). In addition, four questions related to religiosity were included (*‘To what extent do you consider yourself religious*?*’*, *‘How often do you visit a church*, *mosque or religious meeting*?*’*, *‘How often do you pray*?*’* and *‘To what extent do you belief in a God or a higher power*?*’*) and these were all measured on a 7-point Likert scale (1 = *not at all* or *never* and 7 = *very much* or *very often*). Table 1 provides an overview of the descriptive statistics for the first four studies.

**Table 1:** Demographical variables for Study 1 to Study 4.

	*N*	% Female	% Low educated	% Atheist	Religiosity	Age	AQ	EQ	SQ	EQ-SQ	CREDs	Geometrical Figures Video		
												Intentional	Random	Mechanical
Study 1: The Netherlands	65561	54.4	34.5	59.8	2.04 (1.31)	29.5 (11.1)	2.13 (0.38)	-	-	-	-	-	-	-
Study 2: The Netherlands	588	50.9	29.3	49	2.66 (1.84)	39.2 (13.1)	2.41 (0.45)	2.77 (0.55)	-	-	2.27 (2.31)	-	-	-
Study 3: Switzerland	603	78.9	0	27.4	2.56 (1.58)	21.4 (3.8)	2.1 (0.03)	-	-	-	-	-	-	-
Study 4: The USA	797	53.3	41.8	33	3.24 (1.93)	34.6 (10.7)	2.32 (0.33)	2.94 (0.54)	2.7 (0.50)	0.24 (0.68)	3.29 (1.54)	78.5 (20.8)	38.7 (24.5)	25.6 (26.0)

Data are Means with standard deviations between brackets. AQ = Autism Quotient, EQ = Empathy Quotient, SQ = Systemizing Quotient, EQ-SQ = hyper-empathizing, CREDs = Credibility Enhancing Displays.

#### Autism-spectrum quotient

The AQ questionnaire measures participants’ score on traits associated with autism [55]. It consists of 50 items (e.g., *If I try to imagine something*, *I find it very easy to create a picture in my mind*) and all questions were scored on a 4-point Likert scale (‘*definitely agree’*, *‘slightly agree’*, *‘slightly disagree’*, and *‘definitely disagree’*). This is different from the original scale, which scores questions with 0 or 1, but the reliability was comparable (i.e., Cronbah’s alpha [α] = .89 for the 4-point Likert scale instead of α = .86 for the bimodal scale). For the items in which an agree-response was reflective of autistic traits the scoring was reversed. Thus, high AQ scores as well as scores on the AQ subscales (e.g., social skills) were indicative of autistic traits. We used the Dutch version of the AQ, which was translated according to the backward translation procedure [56].

#### Data analysis

To allow comparison with the data obtained in the other countries in later studies, we only examined three of the religiosity questions in the regression model (*‘To what extent do you consider yourself religious*?*’*, *‘How often do you visit a church*, *mosque or religious meeting*?*’* and *‘How often do you pray*?*’)*, reliability α = .84, although we did use all data in a network analysis model which will be explained below. The average religiosity score was highly positively skewed (1.78) and non-normally distributed, Kolmogorov-Smirnov (49105) = .21, *p* < .001. Therefore, religiosity was dichotomized into atheists (average score lower than 2, 59.8%) and believers (average score of 2 or higher, 40.2%). To facilitate comparisons with other countries and because the education-scores were bimodally distributed, Kolmogorov-Smirnov (49105) = .20, *p* < .001, we divided participants in two groups on the basis of a median split (34.5% low educated). To investigate the effect of traits associated with autism on participant’s religiosity, we first conducted generalized linear models for all analyses in the paper. Considering the highly skewed and bimodal distribution of religiosity we first tested a mixture response with Tweedie Log Link (Ma & Jørgensen, 2007) and then divided religiosity in a categorical and subsequently a dichotomous predictor. Because these different analyses did not lead to meaningfully different results, we report the most parsimonious and comprehensible model (i.e., religiosity as a dichotomous predictor). We conducted a hierarchical logistic regression analysis in which the dichotomized religiosity dummy was predicted by the AQ, while controlling for demographic predictors. A hierarchical logistic regression analysis was preferred over a simultaneous model, as some demographical predictors have previously found to be robustly related to religiosity and had to be controlled for [57]. Therefore, in the first step, gender, age and education[58–60] were added as predictors of religiosity using the Enter method (for consistency with other countries, we used this same procedure for all further regression analyses). In the next step, the AQ was included as predictor.

### Results

#### Hierarchical logistic regression analysis

Table 2 shows the outcome of the logistic regression analysis. Compared to the constant only model, the first model was statistically significant, indicating that the predictors reliably distinguished between atheists and theists, χ^2^(3) = 889.55, *p* < .001, although the relationship was weak (.01 = small, .09 = medium, .25 = large) [61], Nagelkerke *R*^*2*^ = .02. Gender and age both made a significant contribution whereas education did not. Females were 1.59 times more likely to be theist than males and with each unit increase in age, the odds of being theist increased with 1.01. In the second model, the AQ was added as predictor. However, the second model was not significant in comparison to the first model, χ^2^(3) = 2.66, *p* = .103, Nagelkerke *R*^*2*^ = .02, indicating that religiosity could not be meaningfully predicted by the AQ.

**Table 2:** Logistic regression analysis for variables predicting religiosity by atheists (N = 29,348) and theists (N = 19,575) in Study 1, controlling for background variables.

	Model 1				Model 2			
	*B*	*SE B*	*eB*	[95% CI]	*B*	*SE B*	*eB*	[95% CI]
Intercept	-1.08	<0.01			-0.99	<0.01		
Gender	0.01*	<0.01	1.01	[1.01–1.02]	0.01*	<0.01	1.01	[1.01–1.02]
Age	0.46*	-0.02	1.59	[1.53–1.64]	0.46*	0.02	1.58	[1.53–1.64]
Education	0.03	-0.02	1.03	[0.99–1.07]	0.03	0.02	1.03	[0.99–1.07]
AQ					-0.04	0.03	0.96	[0.92–1.01]

Gender is coded 1 for females and 0 for males, education is coded 1 for high Educated and 0 for low educated. *eB* = exponentiated B, *B* = odds ratio. AQ = Autism Quotient.

* *p* < .001. *R*^*2*^ (Nagelkerke) = .02 for Model 1 and *R*^*2*^ (Nagelkerke) = .02 for Model 2.

It could be argued that the AQ and the demographical predictors shared some variance, and that by the order in which the predictors were added to the model (i.e., demographical predictors first) there was less variance left for the AQ to explain. An additional analysis in which only the AQ was added revealed that the model reliably distinguished between atheists and theists, χ^2^(1) = 7.18, *p* = .007, although the explained variance of the model was very small, Nagelkerke *R*^*2*^ < .001. To be better able to compare the relative influence of the AQ and the demographical predictors an additional analysis was conducted in which only the demographical predictors were entered. In this model, the predictors at least explained some variance, χ^2^(3) = 889.55, *p* < .001, Nagelkerke *R*^*2*^ = .02.

#### Network model analysis

**Fig 1:**
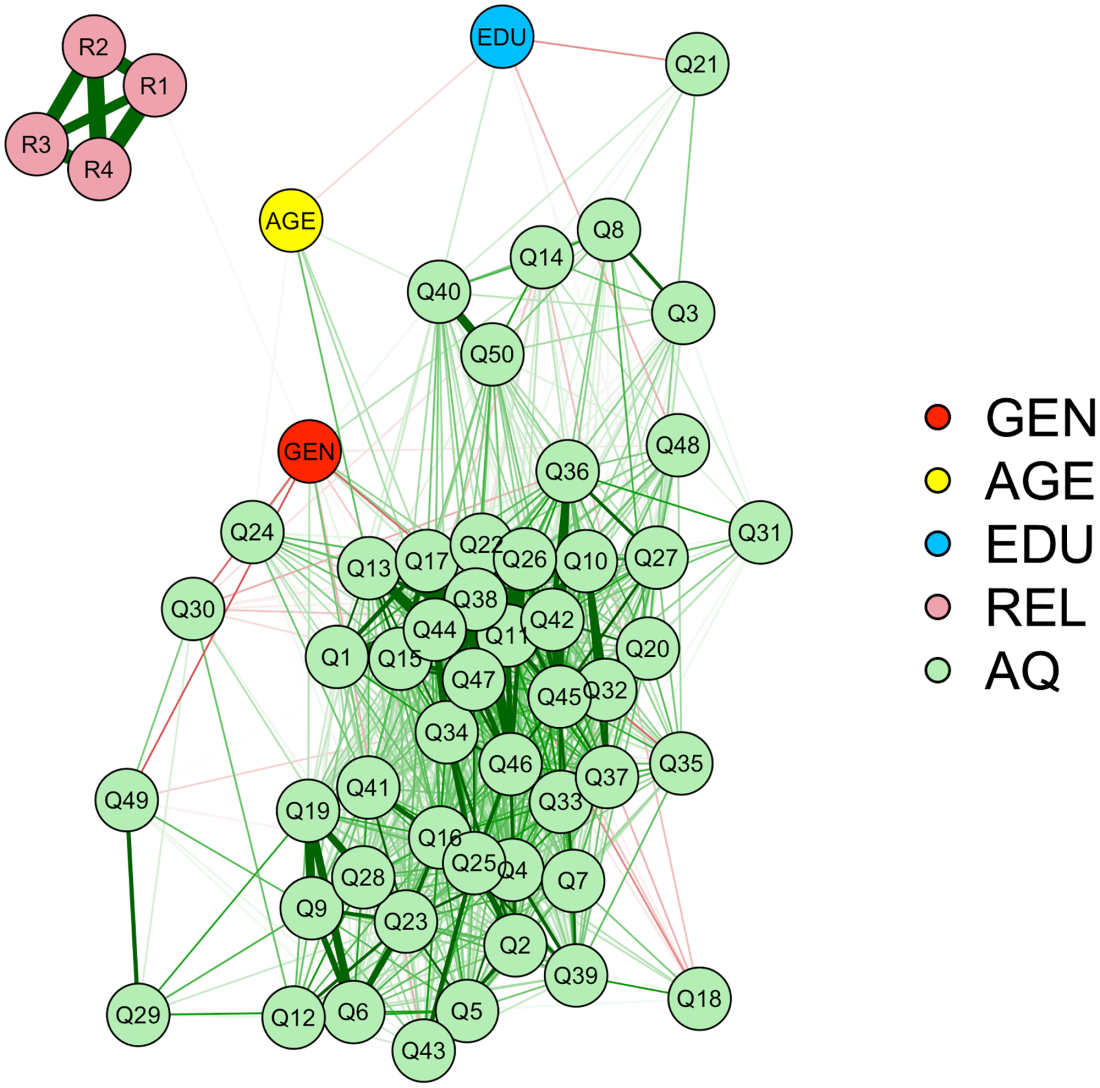
Network analysis model showing a graphical representation of the inter-item correlations among all items used in Study 1. Gen = gender, AGE = Age, EDU = Education, REL = religiosity, AQ = Autism Spectrum Quotient, R1 = God, R2 = praying, R3 = Church, R4 = religiosity, Q1 –Q50 = item 1–50 of the Autism Spectrum Quotient. The lines represent the inter-item correlations. Thicker lines represent larger correlations and correlations are thresholded at *r* = .10. Green lines are indicative of positive correlations, red lines of negative correlations.

The general idea of the supposed relationship between mentalizing and supernatural beliefs is that our mentalizing capacities are a necessary component to be able to represent the intentions of supernatural agents. However, the religiosity questions also tapped into general religiosity and church visit. While it may be logical that mentalizing is related to representing or interacting with a supernatural agent, it may be less logical to suppose a link between mentalizing and visiting churches or religiosity in general. Therefore, in Fig 1 a network analysis model was added [62], showing a graphical representation of the inter-item correlations of all items used in the study. In this way it can be directly investigated whether specific items of the religiosity questionnaire and the AQ are interrelated. According to a clustering algorithm, nodes (i.e., circle in the figure) are placed more closely together when they are more strongly correlated. The threshold for an edge (i.e., line in the figure) to appear between two nodes was a small correlation (*r* > .10; to increase the visibility of the lines the threshold was not constant for all studies). As is evident from the model, none of the religiosity nodes is linked to any of the AQ items, suggesting that there appears to be no relationship between religiosity and the AQ, independent of the specific items used. However, gender was related to belief in God, reflected by the thin line from gender to belief in God (i.e., R1). Please not that this line is relatively thin, reflecting a small correlation (*r* = .10) and may not visible on some screens.

### Discussion

In a large-scale survey, we could not replicate earlier findings that the AQ was a significant predictor of religiosity [16]. The order in which the predictors were added to the model did not have an influence on the interpretation of the results. When the AQ was entered first into the model, none of the variance in religiosity was explained by the AQ. Furthermore, a comparison of a model in which only the AQ was added as predictor and a model in which all demographical predictors were added to the model indicated that the demographical predictors at least explained some variance whereas the significant influence of the AQ was trivial due to the size of the sample. We also showed that it is unlikely that we failed to find an effect due to the way we operationalized religiosity, by adding a network analysis model that graphically visualizes the inter-item correlations between all items used in the model. Even though the threshold of the correlations to appear in the model was set at a fairly low value (a correlation of *r* = .10), no correlations were observed between any of the religiosity items and any of the AQ items. Overall, these results indicate that the relative importance of mentalizing (as assessed with the AQ) for predicting religiosity may be limited.

Apart from cultural differences between the Netherlands and the US that will be addressed in Studies 3 and 4, a concern may be that our sample consisted of a generally highly educated group of people interested in (popular) science (i.e., they were readers of Dutch popular science magazine). However, since previous samples also consisted of highly educated students [16,17] this characteristic of our sample seems unlikely to explain any of the differences between our and previous studies. Further, we doubt that we selectively sampled participants scoring high on the AQ due to our recruitment method (i.e., asking participants to find out how autistic they are) as we had a very large sample and the AQ was normally distributed with comparable means to an earlier study using participants from the Dutch population [56].

What could be considered a limitation is that we only included the AQ as a proxy of mentalizing, while previous studies also used the EQ or the reading the mind in the eye test [16,17,26,63]. Due to the collaboration with the popular science magazine (i.e., Quest), it was only possible for us to request readers to participate in one questionnaire. In Study 2, we addressed this problem by providing readers of the magazine a voluntary option to fill out the EQ as well. In addition, we added a questionnaire on CREDs in order to be able to compare the relative importance of mentalizing in relation to culturally learned aspects of religiosity.

## Study 2: The Netherlands 2

### Introduction

In Study 2, we again investigated whether mentalizing was related to religiosity, this time by taking into account an additional operationalization of mentalizing (i.e., the EQ). In addition, we examined the relative importance of mentalizing in predicting religiosity as compared to CREDs, a specific instance of cultural learning focusing on the credibility of religious actions observed by children during their upbringing.

### Materials and methods

#### Participants

Data were collected from the 5^th^ of January 2015 until the 26^th^ of February 2016 from the same website as reported in Study 1. In total, 15,530 participants filled out the survey. All participants younger than 18 were removed from further analysis (leading to an exclusion of 3,626 participants). Further, we removed all participants who did not fill in all questionnaires (i.e., the additional EQ and religiosity questions; 11,316 participants excluded) and the final dataset consisted of 588 participants. Participants (50.9% female) were on average 29.5 years old (*SD* = 11.1; range 18–85 years), see Table 1 for all demographics.

#### Measures

The measures were the same as in Study 1, except for the addition of two questionnaires: The EQ and a self-constructed version of the Credibility Enhancing Displays scale (CREDs).

#### Empathy quotient

The EQ questionnaire is a scale devised to measure empathy in adults with normal intelligence. It was originally developed by Baron-Cohen and Wheelwright [23] and later abbreviated by Wakabayashi and colleagues [24] to a 22-item scale. All questions were scored on a 4-point Likert scale (‘*definitely agree’*, *‘slightly agree’*, *‘slightly disagree’*, and *‘definitely disagree’*). Half the items were reverse coded to prevent response bias and higher scores were indicative of higher empathy. We used the Dutch version of the EQ, which was translated according to the backward translation procedure [64] with reliability α = .91.

*Credibility Enhancing Displays Scale*. At the time of this study, Lanman and Buhrmester’s CREDs scale [22] was not yet publically available so we constructed seven questions to tap into the concept of CREDs (e.g., ‘*How often did your parents/caretakers attend religious services*?*)*. All other questions can be found in the supplementary material (i.e., the scale had not been validated in earlier Dutch studies, as we were the first to construct these items). All questions were scored on a 7-point Likert scale (1 = ‘*not at all’* to 7 = *‘to a strong extent’*) with a reliability of, α = .81.

#### Procedure

The participant recruitment procedure remained the same as in Study 1. After completing the AQ on the online survey and obtaining their personal AQ score, participants were welcomed to continue with the online survey by the following question: *“We would like to obtain more insight in the relationship between autism and individual differences such as religiosity*. *We would therefore kindly like to ask you to continue with the survey”)*. We do note that the way in which this question to continue the study was framed, with an emphasis on the word ‘religiosity’ instead of all other individual differences that could have been chosen, made it perhaps somewhat more interesting for believers to continue with the study than non-believers. This view was supported by an analysis of variance showing that the extent to which participants believed in God was somewhat higher for participants who continued (*M* = 2.66, *SD* = 1.84; 1 = does not believe at all to 7 = strongly believes) than for participants who only filled out the first part of the survey, consisting of the AQ (*M* = 2.10, *SD* = 1.39), *F*(1, 9294) = 84.54, *p* < .001. Also, the mean religiosity score of Study 2 was slightly higher than in Study 1 (see Table 1 for the demographics of both studies). However, this effect was small (η2 = .01), and compared to the US samples used in previous studies investigating this topic, our sample was still relatively atheistic, so this effect was not likely to have influenced the results.

#### Data analysis

The data analysis was similar to the first study. In the first model, again the demographical predictors were taken as these have been related to religiosity in the past. In the second model the EQ or the AQ was added (correlational analyses showed a strong negative correlation between the two variables, *r* = -.72, *p* < .001, suggesting that it would not be advisable to insert them together), as we wanted to investigate whether variables associated with mentalizing are important for predicting supernatural beliefs. In the third model CREDs were added to explore to what extent cultural learning adds to predicting religiosity in comparison to mentalizing. However, neither the EQ nor the AQ made a significant contribution to the model, so for reasons of brevity we chose to take the EQ and AQ together in the second model. As an explorative analysis, all interaction terms were added to the model but non-significant interactions were dropped for brevity.

### Results

#### Hierarchical logistic regression analysis

Table 3 shows the outcome of the logistic regression analysis. Compared to a constant only model, the first model was statistically significant, indicating that the predictors reliably distinguished between atheists and theists, χ^2^(3) = 11.49, *p* = .009, although the relationship was weak, Nagelkerke *R*^*2*^ = .03. Gender and age both made a significant contribution whereas education did not. Females were 1.55 times more likely to be theist than males and with each unit increase in age, the odds of being theist increased by 1.02.

**Table 3:** Logistic regression analysis for variables predicting religiosity by atheists (N = 288) and theists (N = 300) in Study 2, controlling for background variables.

	Model 1				Model 2				Model 3			
	*B*	*SE B*	*eB*	[95% CI]	*B*	*SE B*	*eB*	[95% CI]	*B*	*SE B*	*eB*	[95% CI]
Intercept	-0.79	0.29			-0.42	1.17			-1.85	1.28		
Gender	0.44**	0.17	1.55	[1.12–2.15]	0.43*	0.17	1.54	[1.10–2.15]	0.54**	0.18	1.71	[1.20–2.43]
Age	0.02*	0.01	1.02	[1.00–1.03]	0.02*	0.01	1.02	[1.00–1.03]	0.03**	0.01	1.03	[1.01–1.05]
Education	0.07	0.18	1.07	[0.75–1.54]	0.09	0.19	1.09	[0.76–1.57]	0.16	0.20	1.17	[0.80–1.71]
AQ					-0.14	0.27	0.87	[0.51–1.47]	-0.05	0.28	0.95	[0.55–1.65]
EQ					-0.01	0.22	0.99	[0.64–1.53]	0.03	0.23	1.03	[0.65–1.61]
CREDs									0.41***	0.07	1.51	[1.32–1.73]
Age18*CREDs									-0.01**	0.01	0.99	[0.99–1.00]

Gender is coded 1 for females and 0 for males, education is coded 1 for high educated and 0 for low Educated. AQ = Autism Quotient, EQ = Empathizing Quotient, CREDs = Credibility Enhancing Displays scale, age18 = age centered at 18 years. *eB* = exponentiated *B*, *B* = odds ratio.

*p < .05.

**p < .01.

***p < .001. *R*^*2*^ (Nagelkerke) < .01 for Model 1 and Model 2, *R*^*2*^ (Nagelkerke) = .14 for Model 3.

In the second model, the AQ and EQ were added as predictors. However, the second model was not significant in comparison to the first model, χ^2^(2) = 0.50, *p* = .777, Nagelkerke *R*^*2*^ = .03. In the third model, CREDs as well as the interaction between CREDs and age (see data analysis) were added as predictors, resulting in a significant contribution to the prediction, χ^2^(2) = 52.65, *p* < .001, Nagelkerke *R*^*2*^ = .14. CREDs and the interaction between CREDs and age (centered at 18 years for ease of interpretation) were both significant predictors. For each unit increase in CREDs, the odds of being theist increased with 1.55. With regard to the interaction effect, age was centered at 18 years, so a one-unit increase in CREDs at the age of 18 decreased the odds of being a theist with 0.99. This indicates that CREDs had a stronger influence on younger participants than on older participants. The demographics did not change much: gender and age still made a significant contribution whereas Education, AQ and EQ did not.

To disentangle the relative contribution of operationalizations of mentalizing (i.e., the AQ and the EQ) from the relative contribution of the demographical predictors and CREDs, we constructed three additional models. In the first model only the AQ and the EQ were entered as predictors, resulting in a non-significant model, χ^2^(2) = 0.88, *p* = .646, Nagelkerke *R*^*2*^ = .002, indicating that our operationalizations of mentalizing did not adequately distinguish atheists from theists. In the second model, only the demographical predictors were entered as predictors, resulting in a significant model, χ^2^(3) = 11.49, *p* = .009, Nagelkerke *R*^*2*^ = .03. In the third model, only CREDs were entered as predictor, resulting in a significant model, χ^2^(2) = 47.71, *p* < .001, Nagelkerke *R*^*2*^ = .10. Thus, a comparison of the explained variance of the models revealed that the relative contribution of both the demographical predictors and CREDs outweighed the relative contribution of mentalizing that seemed to be non-existent for this sample.

#### Network model analysis

**Fig 2:**
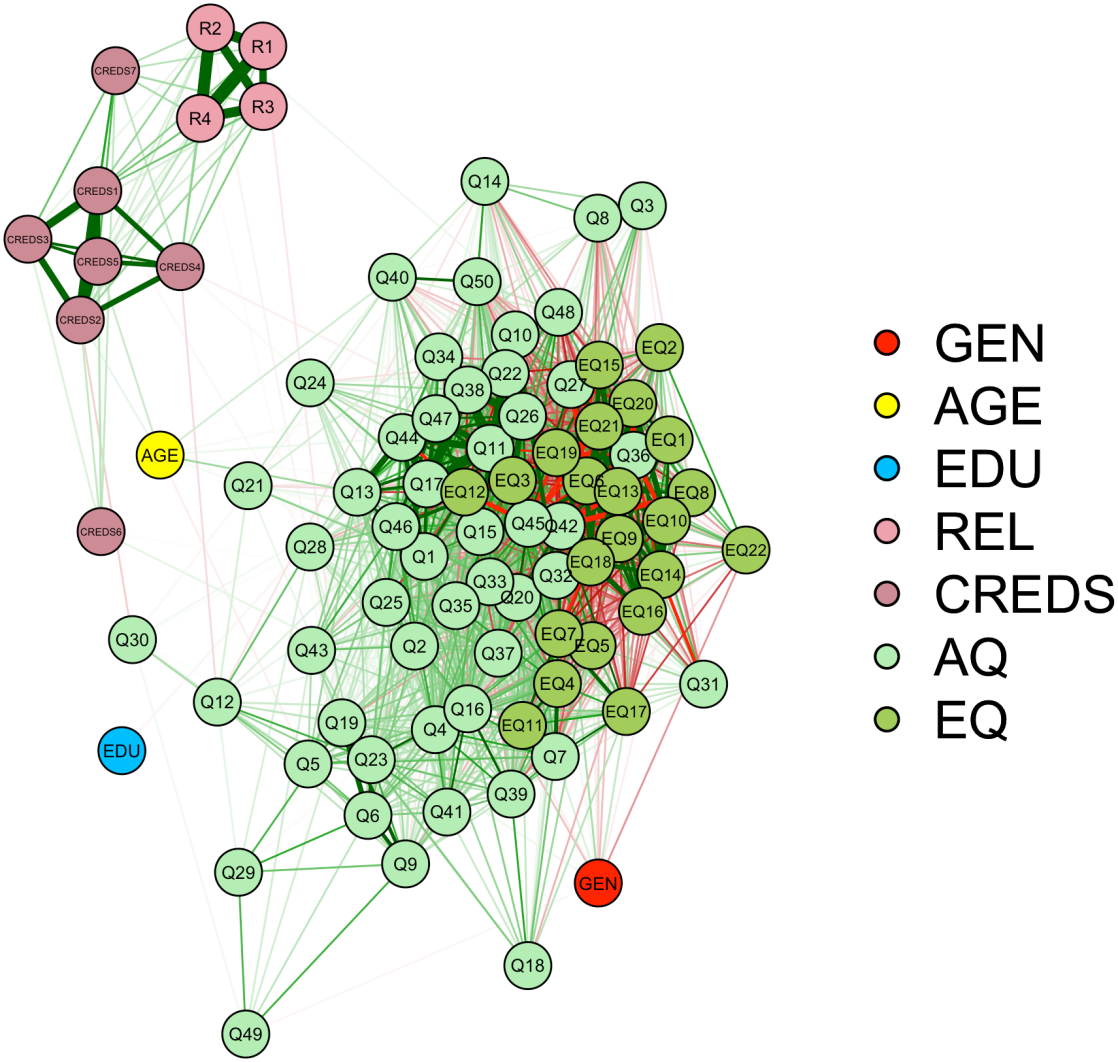
Network analysis entailing a graphical representation of the inter-item correlations among all items used in Study 2. GEN = Gender, AGE = Age, EDU = Education, REL = religiosity, CREDS = Credibility Enhancing Display Scale, AQ = Autism Spectrum Quotient, EQ = Shortened Empathy Quotient, R1 = God, R2 = praying, R3 = Church, R4 = religiosity, CREDS1 –CREDS 7 = item 1–7 of the own-constructed Credibility Enhancing Display Scale, Q1 –Q50 = item 1–50 of the Autism Spectrum Quotient. The lines represent the inter-item correlations. Thicker lines represent larger correlations and correlations were thresholded at *r* = .15. Green lines are indicative of positive correlations, red lines of negative correlations.

Finally, similarly as in Study 1, we conducted a network model analysis to graphically represent the inter-item correlation between all items to rule out the lack of a relationship between mentalizing and religiosity is due to the way religiosity was operationalized. The outcome of the network model analysis is represented in Fig 2 and shows that at least some items of the AQ and EQ were related to religiosity, but that the correlations were weak (lines between nodes were thresholded at *r* > .15). Crucially, the model shows that an absence of a relationship is not likely to be the result of the artificial means with which we formed the construct religiosity, but that it is rather the result of the lack of correlations between the operationalizations of mentalizing and any of the religiosity items.

### Discussion

Again, we did not find the hypothesized relationship between operationalizations of mentalizing (i.e., the AQ or the EQ) and belief in supernatural agents. We did find a strong effect of our self-constructed CREDs scale when predicting religiosity, thereby adding to a growing literature on this topic [20–22,54,65]. We acknowledge that these questions primarily tap into visible markers of religiosity of the parents and are not necessarily equivalent to the central idea of CREDs that ‘actions speak louder than words’ [20,21]. Nevertheless, these findings indicate that i) whether parents’ beliefs are accompanied by credibility-enhancing displays and ii) demographical predictors like age and gender, respectively, are far more important in determining whether people believe than individual differences in mentalizing capacities as assessed with the AQ. Nevertheless, cultural learning is a proximal factor [66] that may explain why people believe and how religiosity spreads; proximal factors do not explain how belief once came into existence (one of the main topics of interest of the cognitive science of religion). For this reason, the fact that in multiple U.S. and Canadian samples in another study [16], mentalizing (as assessed with people with ASD in Study 1, the AQ and EQ in Study 2, Study 3 and Study 4, and the reading the mind in the eye test in Study 4) was a significant predictor of religiosity, is theoretically highly interesting and relevant. Thus, it is important to investigate whether the absence of a role of mentalizing in our studies may be the result of cultural differences between our Dutch samples and the U.S. (and Canadian) samples investigated previously.

An important cultural difference between the U.S. and the Netherlands is that the Netherlands is far more secularized than the U.S. In The Netherlands, only 10% of the believers frequently attend church and The Netherlands have one of the highest percentages of atheists in the Western World [67]. In contrast: in the U.S., 37% of the population frequently attends church [68] and the U.S. has the lowest percentage of atheists of all countries in the Western World [1]. Even the president engages in religious CREDs (e.g., ending each speech by saying *“May God bless you and may God bless the United States of America”*). A possibility is that in highly religious countries fluctuations in mentalizing capabilities (i.e., decreases) can lead to observable effects on religiosity, whereas in highly secular countries fluctuations do not help to explain already prevalent atheism (i.e., a floor effect). Before investigating a religious sample from the U.S. in Study 4, in Study 3, we tried to address this issue by looking at dataset that was available from a study in Switzerland in which all necessary variables were included. Switzerland is a country less secularized than the Netherlands [67,69], but more than the U.S [1].

## Study 3: Switzerland

### Introduction

Switzerland has a moderate to strong interwoven relationship between society and Christianity [69]. The percentage of the population that self-reports to be atheist is almost twice as large in The Netherlands as in Switzerland [1]. Whereas 39–44% of the people reported to be atheist in the Netherlands, this was only true for 17–27% in Switzerland. In this study we investigated the relationship between the AQ and religiosity in a similar fashion as in the first two Dutch studies, although in this dataset the EQ was not taken into account.

### Materials and methods

Data were collected from first year psychology students from the 10^th^ of October 2014 until the 18^th^ of December 2014 at the University of Lausanne. The investigation was part of a larger study validating questionnaires on trait schizotypy and autistic traits [70,71]. In total, 627 participants filled out the survey, but AQ data from one participant was missing. Participants (78.9% female) were on average 21.4 years old (*SD* = 3.8; range 15 to 50 years), see Table 1 for all demographics. The religiosity measure was different from the two studies, with minor changes in terms of the assessed demographics. In the Swiss sample, 15 questions were measured that related to religiosity, however not all participants filled out all these questions. Participants were first asked to answer the question whether they were believer, atheist or agnostic. Second, participants were asked how they defined themselves religiously (i.e., *Christian*, *Jew*, *Muslim*, *Buddhist*, *Hindu*, *Atheist/ not believer*, *agnostic/ we cannot know*, *other*). To be as much consistent with the first studies as possible, we used religiosity (believer vs. atheist) as a dichotomous predictor and left the agnostic people out because agnostics can be either believers or non-believers (leading to an exclusion of 23 participants). The other 13 religiosity items were only filled in by believing and agnostic participants. In the first question, people were asked how often they visited churches and in the third question participants were asked how often they prayed (*rarely or never*, *1–2 times a month*, *more than 2 times a month*). Items 4–13 were measured on a 7 point Likert scale, (1 = *not at all/ not important at all*, to 7 = *strongly/ very important*; e.g., translated from French: ‘*is it easy to represent yourself God or/and his will*?*’*). Further data analyses were similar to Study 1, apart from the predictor ‘education’ that was dropped because all participants were university students.

### Results

#### Hierarchical logistic regression analysis

Table 4 shows the outcome of the logistic regression. The first model was not statistically significant different from a constant only model, indicating that the predictors did not reliably distinguish between atheists and theists, χ^2^(2) = 0.16, *p* = .923, Nagelkerke *R*^*2*^ < .01. In the second model, the AQ was added as predictor. However, the second model was also not significant in comparison to the first model, χ^2^(1) = 2.15, *p* = .143, Nagelkerke *R*^*2*^ = .01.

**Table 4:** Logistic regression analysis for variables predicting religiosity by atheists (N = 240) and theists (N = 168) in Study 3, controlling for background variables.

	Model 1				Model 2			
	*B*	*SE B*	*eB*	[95% CI]	*B*	*SE B*	*eB*	[95% CI]
Intercept	1.13	0.52			-0.04	0.95		
Gender	0.01	0.22	1.01	[0.65–1.57]	0.05	0.23	1.06	[0.68–1.64]
Age	-0.01	0.02	0.69	[0.95–1.04]	-0.01	0.02	0.99	[0.95–1.04]
AQ					0.53	0.37	1.71	[0.83–3.50]

Gender is coded 1 for females and 0 for males. *eB* = exponentiated *B*, *B* = odds ratio. AQ = Autism Quotient. None of the models was significant. *R*^*2*^ (Nagelkerke) < .01 for Model 1 and *R*^*2*^ (Nagelkerke) = .01 for Model 2.

#### Network model analysis

**Fig 3:**
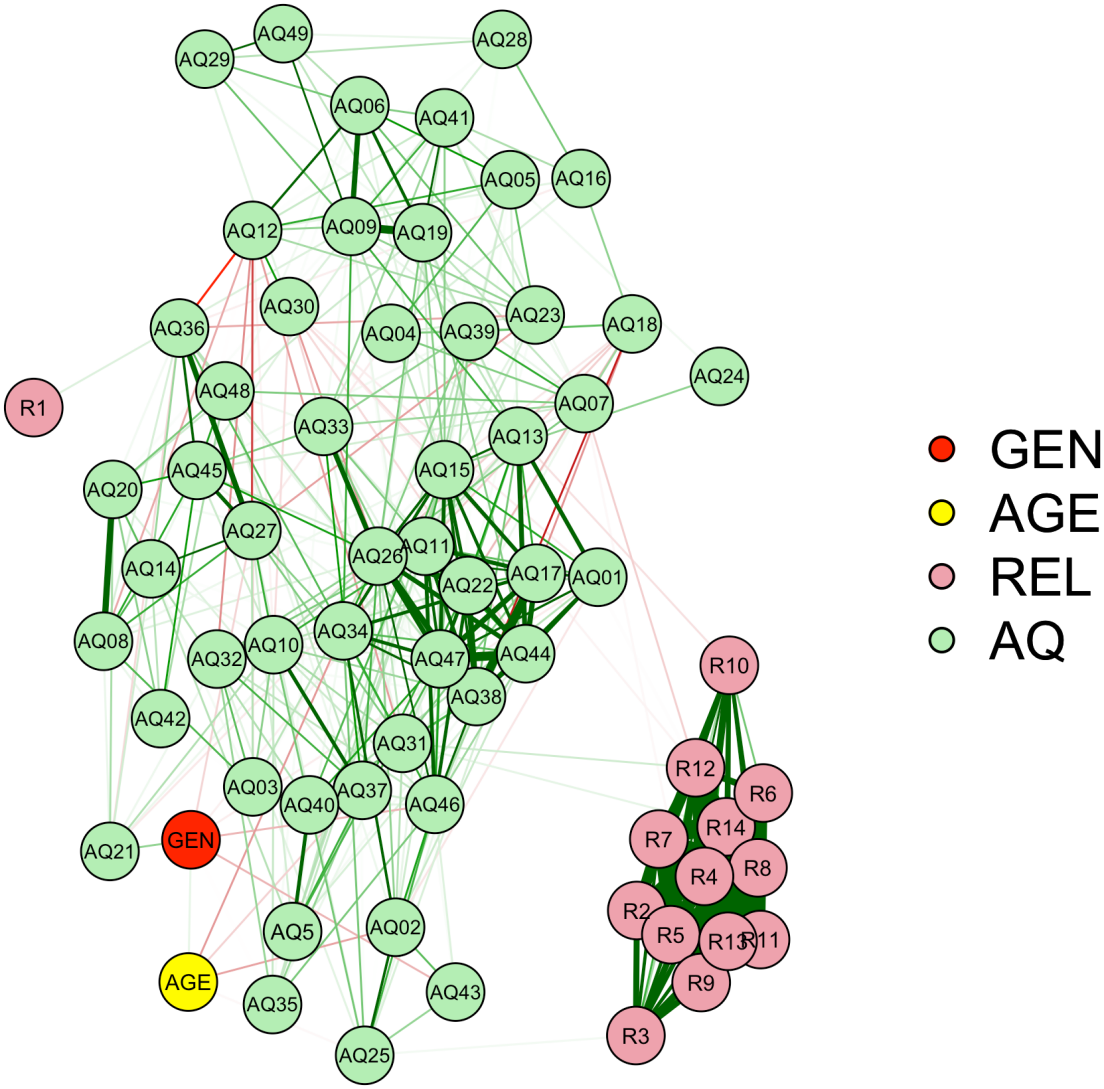
Network analysis entailing a graphical representation of the inter-item correlations among all items used in Study 3. GEN = gender (1 = female, 2 = male), AGE = age, REL = religiosity, AQ = Autism Spectrum Quotient, R1—R14 = religiosity items (see supplementary material online), Q1 –Q50 = item 1–50 of the Autism Spectrum Quotient (see supplementary material online). The lines represent the inter-item correlations, thicker lines represent larger correlations and correlation lines start from *r* = .15. Green lines are indicative of positive correlations, red lines of negative correlations.

Similarly as in the first studies, we conducted a network model analysis to graphically represent the inter-item correlation between all items to rule out that the lack of a relationship between mentalizing and religiosity is due to the way religiosity was operationalized. The outcome of the network model analysis represented in Fig 3 shows that at least some items of the AQ were related to religiosity, but that the correlations were weak (lines between nodes emerged only for *r* > .15). There are more green lines than red lines between the AQ and religiosity items, indicating that the correlations between AQ and religiosity items are more often positive than negative. Crucially, the model shows that an absence of a relationship is not likely to be the result of the means by which we formed the construct religiosity (believing yes/no), but that it is rather the result of the lack of the strength of the correlations between the AQ and any of the religiosity items.

### Discussion

In Study 3, we investigated the possibility that in highly religious countries fluctuations in mentalizing capabilities (i.e., decreases) can lead to observable effects on religiosity, whereas in highly secular countries they do not add to explain the already prevalent atheism. However, we did not find the presumed relationship between the AQ and religiosity in a sample from a less secularized country than the Netherlands. The percentage of atheists was lower in the Swiss sample than both Dutch samples. Thus, it is unlikely that we failed to observe an inverse relationship between religiosity and the AQ in The Netherlands due to the high secularity in this country. Moreover, in the network analysis model all measured religiosity items were taken into account and the AQ items were more often positively related with religiosity rather than negatively.

Two differences between the Dutch and the Swiss sample are important to note. First, the Swiss sample was more highly educated than both of the Dutch samples as all Swiss participants were university students. On the one hand, it may seem remarkable that the percentage of theists was higher although analytic thinking [72] as well as intelligence [73] have repeatedly been related to disbelief. On the other hand, the effects of analytic thinking and intelligence were weak and the previous studies from the current paper as well as other studies [16,72] have shown that education may not be a robust predictor when it comes to explaining religiosity when factors as gender and age are being controlled for.

Second, the percentage of females was considerably higher in the Swiss sample than in the Dutch samples, a factor that could also explain the higher percentage of theists in the Swiss sample since the experiments above and previous studies have shown that females more strongly believe than males [16,58,72]. Further, females score lower on the AQ than males [55], possibly diminishing the potential influence of individual differences in the AQ on predicting religiosity. Speaking against this, however, is the finding that the AQ score was somewhat higher in the Swiss sample than in the Dutch samples, probably due to the fact that the Swiss sample consisted of only high-educated participants and highly educated people score higher on the AQ than lower educated participants [55]. Nevertheless, while Switzerland may be less secularized than The Netherlands, there are still large differences between Europe and the United States, as the United States is one of the most religious countries of the Western World [1]. Thus, there may be cultural differences between the United States and Europe that may explain why a relationship between mentalizing and religiosity is more present in the USA than in Europe. To address this issue we conducted a direct replication of the study of Norenzayan et al. [16] by recruiting a group of participants from the US. We pre-registered this study on the Open Science Framework (https://osf.io/6vrne/).

## Study 4: United States of America

### Introduction

In Study 4, we conducted a replication of the study of [16] by using a sample from the U.S. as well as using the exact materials as provided by of one of the co-authors of that study (i.e., Will Gervais). This means that we used the same material as in Study 2 and added the systemizing quotient (SQ) and several religiosity items. The SQ measures the drive to analyze or construct systems. This scale was not related to religiosity in the study of Norenzayan et al. [16]. It has been suggested, however, that not mentalizing, but hyper-empathizing is predictive of religiosity [25]. The underlying idea is that humans have two parallel cognitive systems, one for mentalizing (i.e., interaction with the psychological environment) and one for systemizing (interaction with the physical environment)[74,75]. Specifically the combination of high empathizing (good mentalizing capacities) and low systemizing (poor understanding of how the physical world works) may encourage supernatural beliefs. Thus, adding the SQ allowed us to more directly replicate the study of Norenzayan et al. (16) as well as to test the hypothesis that hyper-empathizing predicts religiosity as has been suggested and found in earlier studies [63].

Furthermore, we wanted to tap into the concept of mentalizing ability differently by using an experimental measure of mentalizing ability (i.e., not relying on self-report questions with validity problems that have been outlined in the introduction). Therefore, we added the geometrical figures task (GFT). In this task, participants watch geometrical figures move as if they have goal directed intentions (i.e., the figures chase each other). In line with the proposed theory that mentalizing deficiencies decrease religiosity, we predicted that decreased intentionality ratings on the videos would be associated with decreased religiosity.

### Materials and methods

#### Participants

Data were collected from the 4^th^ of November 2015 until the 16^th^ of January 2016 on Amazon’s Mechanical Turk in which we aimed to test approximately 250 atheists, 250 spiritual and 250 Christian believers to obtain sufficient variability in religiosity for another study. In total, 1.235 participants started the survey, and of which 797 participants (53.3% female) completed it (64.5% completion rate; *M* age = 34.6, *SD* = 10.7, range 18 to 70). Participants received $2.50 for participation.

#### Measures and procedure

On the website of Amazon Mechanical Turk, participants were offered the opportunity to conduct an online survey. The first question required participants to indicate the kind of belief system they endorsed (“*non-believer/atheist*, *Christian*, *Muslim*, *Hindu*, *Spiritual believer*, or *another belief system”)*. If participants reported not to consider themself an atheist, Christian or spiritual believer, they were directed to the end of the survey. To prevent people from participating twice, people could not participate with the same IP-address more than one time. The following questionnaires were obtained in respective order: demographics (age, gender, social economical status, years of education), religiosity (although we used the exact same questions as used in Norenzayan et al. [16] we only analyzed the questions that were also obtained in Study 1 and Study 2 to ease comparison between countries), α = .89, CREDs (as measured with Lanman and Burhmester, [22] scale), α = .92, AQ, α = .86, EQ, α = .83, the systemizing quotient (SQ), α = .88 and the Geometrical Figures Task.

#### Systemizing quotient

The SQ measures the drive to analyze or construct systems. It was first developed by Baron-Cohen [76], later abbreviated by Wakabayashi et al. [24] and consists of 25 items on which participants could either agree or disagree (e.g., *“I am fascinated by how machines work”* and *“I find it difficult to read and understand maps”*), some of which were reverse-scored. Higher scores were indicative of higher self-reported systemizing skills, α = .83.

#### Credibility enhancing displays

As explained above, CREDs are signals (i.e., displays) of actions that increase or decrease the likelihood of believing in the existence of the supernatural [21]. We here used Lanman and Buhmester’s validated CREDs scale [22]; e.g., *Overall*, *to what extent did your caregiver(s) act as good religious role models*?”). All questions were scored on a 7-point Likert scale (1 = ‘*not at all’* to 7 = *‘to a strong extent’*) with reliability α = .92.

#### Geometrical figures task

We used an adapted version of the Geometrical Figures Task developed by Riekki, Lindeman and Raij [51] in which animations displayed moving geometrical figures. Participants had to rate to what extent movements performed by the geometrical figures were intentional by adjusting a scale from 1 (*no intentionality present*) to 100 (*strong intentionality present*). Participants were first shown three practice videos, one of each category (i.e., intentionality, mechanically and random). Each practice video was accompanied by an instruction explaining why the video was intentional or non-intentional (mechanic or random). For the intentional movements video it was explained that the figures moved as if they had an intention, for example as if one figure chased the other. For the mechanical video, it was explained that the figures moved as if following the laws of physics. So, if one figure touched another the other figure would also start moving or if the figure touched the wall it would bounce back. The random movements were semi-random as the animations were programmed in such a way that figures would not touch each other, otherwise figures would appear to run through each other. Participants were instructed that these figures moved randomly and that there was no logical mechanical or intentional pattern observable in the movements of the figures.

The stimuli of Riekki et al. [51] were developed for a functional magnetic resonance imaging study and therefore quite easy to rate in terms of intentionality and randomness. In order to increase the difficulty (and ambiguity) we cut the original videos of 30 seconds in 3 parts of 10 seconds. In addition, we increased the speed of the videos by changing the length to 6 seconds per video. In total, we used 24 clips, 8 of each video type (i.e., intentional, random and mechanical motion). Each participant rated only a pseudo-randomized subset of 9 videos (3 from each video type).

#### Data analysis

The logistic analyses were similar to the previous studies: religiosity was non-normally distributed, Kolmogorov-Smirnov (787) = .09, *p* < .001. Therefore, religiosity was dichotomized into atheists (average score lower than 2, 33%) and theists (average score of 2 or higher, 67%). In the first model the demographical variables were taken as predictors. In the second model all operationalizations of mentalizing were added: the AQ, EQ-SQ and the GFT. In the final model, CREDs were added. As an explorative analysis, all interaction terms were added to the model but non-significant interactions were dropped for conciseness.

### Results

#### Hierarchical logistic regression

Compared to a constant only model, the first model was statistically significant, indicating that the predictors reliably distinguished between atheists and theists, χ^2^(3) = 35.17, *p* < .001, Nagelkerke *R*^*2*^ = .06 (see Table 5 for the outcomes of the logistic regression analysis). Gender and age both made a significant contribution whereas education did not. For females, the odds were 1.78 times more likely to be theist than for males and with each unit increase in age, the odds of being theist increased with 1.03. In the second model, all operationalizations relating to mentalizing (i.e., the AQ, EQ-SQ and the GFT) were added as predictors to the model and they significantly contributed to the model, χ^2^(5) = 33.40, *p* < .001, Nagelkerke *R*^*2*^ = .12. Seeing intentionality in random videos as well as mechanistic videos made a significant contribution to the model, whereas the AQ, hyper-systemizing, and seeing intentionality on intentional videos did not. With each unit increase on the random video as well as on the mechanistic video (scale = 1–100), the odds were 1.01 times more likely to be theist than atheist. The other predictors did not change much in comparison to the first two models: gender and age still made a significant contribution.

**Table 5:** Logistic regression analysis for variables predicting religiosity by atheists (N = 263) and theists (N = 524) in Study 4, controlling for background variables.

	Model 1				Model 2				Model 3			
	*B*	*SE B*	*eB*	[95% CI]	*B*	*SE B*	*eB*	[95% CI]	*B*	*SE B*	*eB*	[95% CI]
Intercept	-0.67	0.29			-1.24	0.52			-2.19	0.56		
Gender	0.58***	0.17	1.78	[1.31–2.43]	0.50**	0.17	1.64	[1.17–2.30]	0.52**	0.18	1.68	[1.19–2.37]
Age	0.03***	0.01	1.03	[1.02–1.05]	0.04***	0.01	1.04	[1.02–1.06]	0.03***	0.01	1.03	[1.02–1.05]
Education	0.10	0.18	1.11	[0.81–1.51]	0.02	0.16	1.02	[0.74–1.40]	0.06	0.17	1.07	[0.77–1.48]
AQ					-0.21	0.29	0.81	[0.46–1.43]	-0.06	0.36	0.95	[0.53–1.70]
EQ-SQ					0.26*	0.15	1.30	[0.97–1.75]	0.29	0.18	1.33	[0.98–1.81]
Intentional					0.00	0.01	1.00	[0.99–1.01]	0.00	0.01	1.00	[0.99–1.01]
Random					0.01*	0.00	1.01	[1.00–1.01]	0.01*	0.00	1.01	[1.00–1.02]
Mechanistic					0.01**	0.00	1.01	[1.00–1.02]	0.01**	0.00	1.01	[1.00–1.02]
CREDs									0.38***	0.06	1.46	[1.30–1.63]

Gender is coded 1 for Females and 0 for Males, education is coded 1 for High Educated and 0 for Low Educated. AQ = Autism Quotient, EQ = Empathizing Quotient, EQ-SQ = hyper-systemizing, intentional, random, and mechanistic are the different intentionality ratings for the geometrical figures videos, CREDs = Credibility Enhancing Displays scale. *eB* = exponentiated *B*, *B* = odds ratio.

*p < .05.

**p < .01.

***p < .001. *R*^*2*^ (Nagelkerke) = .06 for Model 1, *R*^*2*^ (Nagelkerke) = .07 for Model 2, *R*^*2*^ (Nagelkerke) = .12 for Model 3 and *R*^*2*^ (Nagelkerke) = .19 for Model 4.

In the third model, CREDs were added resulting in a significant contribution, χ^2^(1) = 47.91, *p* < .001, Nagelkerke *R*^*2*^ = .19. With each unit increase on the CREDs scale (1 to 7) the odds of being theist increased with 1.46. The other predictors did not change much in comparison to the first models: gender, age and the random and mechanical videos still made a significant contribution whereas education, the AQ and hyper-systemizing did not.

To disentangle the relative contribution of operationalizations of mentalizing (i.e., the AQ, SQ-EQ and the GFT) from the relative contribution of the demographical predictors and CREDs, we constructed three additional models. In the first model the AQ, EQ and GFT were entered as predictors, resulting in a significant model, χ^2^(3) = 14.08, *p* = .001, Nagelkerke *R*^*2*^ = .03, indicating that our operationalizations of mentalizing did distinguish atheists from theists. In the second model, only the demographical predictors were added, resulting in a significant model, χ^2^(3) = 35.17, *p* < .001, Nagelkerke *R*^*2*^ = .06. In the third model, only CREDs were entered as predictor, resulting in a significant model, χ^2^(1) = 60.60, *p* < .001, Nagelkerke *R*^*2*^ = .10. Thus, a comparison of the explained variance of the models revealed that the relative contribution of both the demographical predictors and CREDs outweighed the relative contribution of our operationalizations of mentalizing.

**Fig 4:**
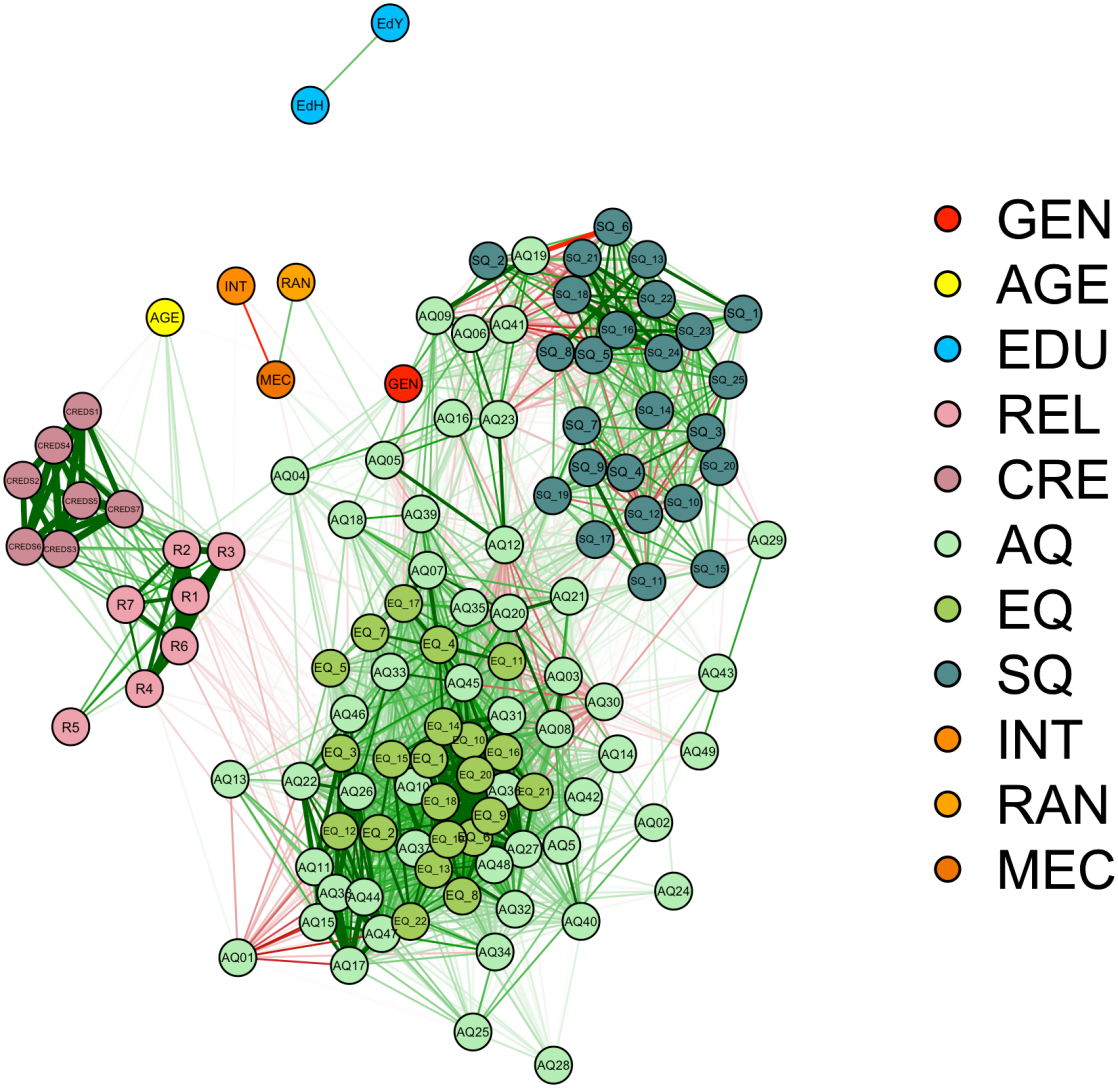
Network analysis entailing a graphical representation of the inter-item correlations among all items used in Study 4. GEN = gender (1 = female, 2 = male), AGE = age, EDU = education, REL = religiosity, CRE = CREDs (i.e., Credibility Enhancing Displays scale), AQ = Autism Spectrum Quotient, EQ = Empathy Quotient, SQ = Systemizing Quotient, INT = intentionality rating for the intentionally moving geometrical figures, RAN = intentionality rating for the random moving geometrical figures, MEC = intentionality rating for the mechanically moving geometrical figures, R1—R14 = religiosity items (see supplementary material online), Q1 –Q50 = item 1–50 of the Autism Spectrum Quotient (see supplementary material online). The lines represent the inter-item correlations, thicker lines represent larger correlations and correlation lines start from *r* = .15. Green lines are indicative of positive correlations, red lines of negative correlations.

Finally, similar to the previous studies we conducted a network model analysis to graphically represent the inter-item correlation between all items. The outcome of the network model analysis is represented in Fig 4 and shows that several items of the AQ and EQ were related to the religiosity items (lines between nodes were thresholded at *r* > .15). Essentially, the model shows that there are multiple correlations between the AQ, EQ and SQ items on the one hand and religiosity items on the other hand. Importantly, most of these relationships are negative and thus in line with the notion that reduced ToM capacities are linked to reduced belief in supernatural agents.

#### Explorative analysis: Curvilinear relation between AQ and religiosity

A still open-standing possibility is that the relationship between the AQ and belief in supernatural agents might better be captured by a curvilinear relationship than by a linear relationship, perhaps explaining the lack of the fit of the AQ in the previous models. The underlying idea is that for people with high scores on the AQ it may be problematic to represent supernatural agents or read the intentions of supernatural agents, whereas for people scoring low to moderate on the AQ, no relationship would be expected (resulting in a random distribution). To investigate this possibility a logistic regression model was conducted similar to the first model of Study 4, except for the fact that the quadratic term was added to account for a possible curvilinear effect [77]. In order to do so, the AQ was centered (i.e., AQ-centered) and added to the model and the quadratic term of the centered predictor (i.e., AQ^2^-centered) was also added to the model.

The outcomes of the first model are identical to the first model of Study 4. In the second model all operationalizations relating to mentalizing (i.e., the AQ-centered, AQ^2^-centered, EQ-SQ and the GFT) were added as predictors to the model and they significantly contributed to the model, χ^2^(6) = 50.87, *p* < .001, Nagelkerke *R*^*2*^ = .14 (see Table 6). The quadratic term of the AQ as well as attributing intentionality to both the random and mechanistic videos all significantly added to the model. For each unit increase on the quadratic term of the centered AQ, the odds were 6.99 (i.e., 1/0.143) more likely to be theist than atheist. For each unit increase on the random and mechanical video, the odds of being theist increased with 1.01. The other predictors did not change much in comparison to the first model: gender and age still made a significant contribution.

**Table 6:** Explorative logistic regression analysis for variables predicting religiosity by atheists (N = 263) and theists (N = 524) in Study 4, controlling for background variables.

	Model 1				Model 2				Model 3			
	*B*	*SE B*	*eB*	[95% CI]	*B*	*SE B*	*eB*	[95% CI]	*B*	*SE B*	*eB*	[95% CI]
Intercept	-0.67	0.29			-1.14	0.53			-2.06	0.56		
Gender	0.58***	0.17	1.78	[1.31–2.43]	0.58***	0.18	1.78	[1.26–2.51]	0.60***	0.18	1.82	[1.23–2.59]
Age	0.03***	0.01	1.03	[1.02–1.05]	0.04***	0.01	1.04	[1.03–1.06]	0.04***	0.01	1.04	[1.02–1.05]
Education	0.10	0.18	1.11	[0.81–1.51]	-0.03	0.16	0.98	[0.71–1.35]	0.03	0.17	1.03	[0.74–1.43]
AQ-centered					-0.16	0.29	0.85	[0.48–1.51]	-0.02	0.30	0.99	[0.55–1.77]
AQ^2^-centered					-1.95***	0.49	0.14	[0.06–0.37]	-1.88***	0.51	0.15	[0.06–1.41]
EQ-SQ					0.24	0.15	1.27	[0.94–1.71]	0.26	0.16	1.30	[0.96–1.77]
Intentional					0.00	0.01	1.00	[0.99–1.01]	0.00	0.01	1.00	[0.99–1.01]
Random					0.01*	0.00	1.01	[1.00–1.01]	0.01*	0.00	1.01	[1.00–1.01]
Mechanistic					0.01**	0.00	1.01	[1.00–1.02]	0.01**	0.00	1.01	[1.00–1.02]
CREDs									0.37***	0.06	1.45	[1.30–1.62]

Gender is coded 1 for females and 0 for males, education is coded 1 for high educated and 0 for low educated. AQ-centered = centered Autism Quotient, AQ2-centered = quadratic term of the centered Autism Quotient, EQ-SQ = hyper-systemizing, intentional, random, and mechanistic are the different intentionality ratings for the geometrical figures videos, CREDs = Credibility Enhancing Displays scale. *eB* = exponentiated *B*, *B* = odds ratio.

*p < .05.

**p < .01.

***p < .001. *R*^*2*^ (Nagelkerke) = .06 for Model 1, *R*^*2*^ (Nagelkerke) = .07 for Model 2, *R*^*2*^ (Nagelkerke) = .12 for Model 3 and *R*^*2*^ (Nagelkerke) = .19 for Model 4.

In the third model, CREDs were added to the model resulting in a significant contribution, χ^2^(1) = 45.69, *p* < .001, Nagelkerke *R*^*2*^ = .22. With each unit increase on the CREDs scale (1 to 7) the odds of being theist increased with 1.45. The other predictors did not change much in comparison to the first models: gender, age, the quadratic term of the centered AQ and the random and mechanical videos still made a significant contribution whereas education, the centered AQ and hyper-systemizing did not.

To be able to disentangle the relative contribution of all operationalizations of mentalizing (i.e., the AQ-centered, AQ^2^-centered, SQ-EQ and the GFT) from the relative contribution of the demographical predictors and CREDs, we constructed two additional models. In the first model only the operationalizations of mentalizing were entered as predictors, resulting in a significant model, χ^2^(6) = 44.04, *p* < .001, Nagelkerke *R*^*2*^ = .08, indicating that our operationalizations of mentalizing distinguished atheists from theists. In the second model only the quadratic term of the centered AQ was entered as predictor, resulting in a significant model, χ^2^(1) = 14.59, *p* < .001, Nagelkerke *R*^*2*^ = .03, again indicating that the quadratic term of the centered AQ distinguished atheists from theists. Above, we already showed that the explained variance of the demographical predictors was Nagelkerke *R*^*2*^ = .06, whereas the explained variance of the CREDs was Nagelkerke *R*^*2*^ = .10. This indicates that in the US sample the operationalizations of mentalizing were somewhat less important than CREDs, but comparable to the demographical predictors gender and age.

### Discussion

In the fourth study, we could explain 19–22% of the variance in religiosity by means of just two demographical variables (i.e., gender and age) and two constructs (i.e., all mentalizing operationalizations and CREDs). The findings of the studies above were partially replicated: CREDs, age and gender significantly predicted religiosity, whereas the AQ and hyper-systemizing did not. Extending the studies above, we observed that attributing intentionality to mechanical or random videos did account for some of the variance in religiosity.

Explorative analyses revealed that it may be the case that specifically high scores on the AQ are linked to decreased belief in supernatural agents, whereas no such relationship was present for lower scores (i.e., an inverted hockey stick shape). We ruled out that this was the result of adding a quadratic term in general: for none of the other predictors we found a significant contribution when the centered quadratic term was added, even not for the CREDs. Following these outcomes, we also fitted curvilinear models on all other studies (i.e., Study 1–study 3), but this did not result in similar findings; the quadratic term was not related to religiosity in the Dutch or Swiss sample. Thus, this relation may be considered a false positive, or there is a cultural difference explaining why we observed a curvilinear relationship between the AQ and religiosity in the U.S. but not in the other countries (e.g., due to a floor-effect of religiosity in some countries, the association between mentalizing and religiosity does not become apparent).

Attributing intentionality to random and mechanistic videos significantly contributed to predicting religiosity. Thus, when participants reported to perceive more intentionality in moving geometrical figures in which intentionality was absent, they also more strongly endorsed religious beliefs. Similar findings were obtained by van Elk [78] and Riekki et al. [51], who observed that paranormal believers attributed more intentionality to random moving geometrical figures than skeptics. The findings that over-attribution of intentionality is predictive of supernatural beliefs is in line with the idea of a hyperactive agency detection [4] or intentionality ‘device’ [79]. According to these ideas, over-attribution of agency (i.e., seeing intentionality where it is objectively not present) encourages people to belief in supernatural intentionality. Considering that the data are correlational, it could also be the case that people who have been raised religiously and learned to perceive intentionality (e.g., God’s will) in coincidental events are more sensitive to perceive intentionality in ambiguous situations.

The videos were added to be able to tap more directly into mentalizing abilities than the self-report measures (EQ, SQ and AQ) used in earlier studies [16,17,25], but perceiving intentionality in the videos was not related to the questionnaires. For scales that are used for their indirect association with the ability to mentalize, the absence of a relationship with a task that is used to localize mentalizing in the brain seems at least undesirable. On the one hand, these findings may add to comments of other researchers who have questioned the validity of using the EQ as operationalization of mentalizing capacities [17,27,80]. On the other hand, these findings may just suggest that operationalizations of somewhat different constructs were used. Whereas the AQ and EQ may tap into the self-reflected mentalizing ability of people, the outcomes on the GFT may be rather a reflection of implicit mentalizing abilities, or the result of deliberate systemizing skills (see the introduction and discussion of Study 4).

With regards to hyper-empathizing, our findings deviate from earlier suggestions of Baron-Cohen et al. [75] and observations of Lindeman et al. [25] who suggested that rather than high systemizing or low mentalizing alone, the specific combination of high mentalizing and weak systemizing skills may encourage religiosity. Our findings add to this literature by showing that if anything, the relative contribution of this cognitive bias in the way we operationalized is small when compared to the relative importance of demographical variables like gender and age or cultural learning factors such as CREDs. However, other researchers [81–83] have pointed out that the empathizing/systemizing dichotomy insufficiently captures the two parallel modes of cognition that humans have evolved (i.e., mentalistic cognition and mechanistic cognition). Especially systemizing is criticized for being a too narrow construct, as it is restricted to understanding the behavior of systems, whereas mechanistic cognition incorporates this as well as it basically extends to the entire physical world. Thus, future research should try to better capture these modes of cognition, to investigate its relationship to supernatural beliefs.

Further, the network analysis showed that for the US sample far more items of the AQ were related to religious beliefs than for the first three studies. This indicates that in general, the correlations between religiosity and the AQ items were somewhat higher than in the Netherlands and Switzerland. This suggests that the differences may be the result of cultural differences. One problem of the previous studies in the current paper is that all the operationalizations of mentalizing used have limitations, making it unclear to what extent the construct mentalizing was captured. Therefore, in the final study a group of people with ASD (i.e., a group with mentalizing deficiencies) was compared to a group of people without ASD (see the introduction for a discussion of previous studies on this topic).

## Study 5: The Netherlands 3

### Introduction

Similar to Study 1 of Norenzayan et al. [16], we investigated whether people with mentalizing problems (i.e., people with an ASD diagnosis) are less inclined to endorse supernatural beliefs. To investigate this, we compared adolescents with an ASD diagnosis to adolescents without such a diagnosis in terms of their religiosity, religious behaviors and CREDs. Religious behaviors (e.g., praying, ritualized behaviors) were also taken into account because we speculated that the way in which people with ASD engage in religiosity might still be high but rather different (i.e., with a focus on ritualized behavior instead of beliefs). For example, Swanson [39] proposed that children may be able to come to know God via ritual (i.e., behavioural) practices in religion. CREDs were taken into account to rule out that any between group differences in religiosity were the result of stronger religious upbringing. On the basis of the curvilinear relationship observed in the U.S. sample (i.e., Study 4) we predicted that a group of people with ASD would have significantly lower supernatural beliefs than people without an ASD diagnosis. Further, we speculated that if people with ASD would engage in religiosity, then this would be rather reflected primarily in religious behaviors rather than religious beliefs. Finally, we predicted that autistic people would overall attribute less intentionality to videos of the GFT, but that this would be especially evident in the videos in which intentionality was present.

### Materials and methods

#### Participants

Data were collected from two nearby high schools (2 kilometers in distance) in the center of Rotterdam. One high school (Heer Bokel College) was specialized in educating adolescents with ASD, the other was a regular high school (Wolfert van Borselen) but we recruited adolescents from the same educational level (i.e., HAVO: The Dutch equivalent of the senior general secondary education). We recruited 34 participants at the high school for adolescents with ASD but one did not have an official ASD diagnosis and was dropped from further analyses (for the descriptive statistics of both groups, see Table 1). Specifically, 8 adolescents were diagnosed with classical ASD, 13 with Asperger’s syndrome, 15 with pervasive developmental disorder—not otherwise specified, 1 with multiple-complex developmental disorder, 2 with a social communication disorder and of 4 we could not obtain the specific diagnosis. In addition, these disorders were sometimes accompanied by attention deficit hyperactivity disorder (16.2%) or attention deficit disorder (13.2%). We recruited 30 control participants but one participant had an ASD diagnosis and was dropped from further analyses (we did not add this participant to the ASD group as we reasoned that the severeness of ASD may have been weaker considering that the participant went to a general high school). Significantly more males were recruited in the group with ASD (28) than in the control group (17), χ^2^(1) = 5.34, *p* = .021, Cramer’s *V* = 0.29, which is in accordance with previous literature [55,84], but the groups did not differ in age (range 13–18 years, ASD group *M* = 14.6, *SD* = 1.4; NO ASD group = 14.5, *SD* = 1.3), Welch’s *t* (59.8) = 0.27, *p* = .787, *d* = 0.07. Participants received confectionery and fruit for participating in the survey. The adolescents as well as the parents signed informed consent and the Ethical Committee of the University of Amsterdam approved the study. With regard to the ‘capacity’ of people with ASD to provide consent, it is important to note that all participants were high-functioning individuals on a high educational level.

#### Measures

We used the same materials as in the earlier studies: the AQ (α = .84), GFT (intentional, random and mechanic videos; reliabilities are not available as not all videos were seen by all participants), religiosity (α = .84) and CREDs (α = .74). In addition, a self-constructed and unvalidated religious behavior scale was added consisting of 4 items (i.e., *How often do you engage in the following religious activities*: *praying*, *meditation*, *religious ceremonies*, *ritualized behaviors*) on a 7-point Likert scale (1 = never, 7 = very often). The reliability was accurate, α = .86.

#### Procedure

Participants had to report their demographical variables and filled in the religiosity questionnaires and the CREDs scale. Subsequently, participants were instructed about the GFT (see Study 4 for a detailed description of this task). They were shown three practice videos; one of each category (i.e., intentional, random and mechanical). In total, we used 24 clips, 8 of each video type (i.e., intentional, random and mechanical motion). Each participant rated only a pseudo-randomized subset of 15 videos (5 from each video type). Finally, participants filled in the AQ.

#### Data analysis

To investigate whether adolescents with ASD differed from adolescents without ASD on the AQ, religiosity, religious behaviors, CREDs and the GFT videos (i.e., intentional, mechanical and random) we conducted a series of independent samples Welch’s *t*-tests and all significance levels were set at .05 (i.e., two-tailed).

### Results

As expected, adolescents with ASD diagnoses scored higher on the AQ than adolescents without such a diagnosis, Welch’s *t* (60) = 2.89, *p* = .005, *d* = .73 (see Table 7 for *M*’s and *SD*’s). With regards to the religiosity measures, in contrast to our expectations the groups did not differ on religiosity, *t*(59.8) = 0.23, *p* = .819, *d* = 0.06, religious behaviors, *t*(57.2) = 0.21, *p* = .836, *d* = 0.05, or CREDs, *t*(59.5) = 0.96, *p* = .340, *d* = 0.24. With regards to the GFT videos, we found that adolescents with ASD ascribed less intentionality towards random, *t*(59.1) = 2.14, *p* = .036, *d* = 0.55 and mechanical videos, *t*(51.0) = 2.79, *p* = .007, *d* = 0.72, than adolescents without ADS, but no difference was observed for the intentional videos, *t*(56.8) = 1.12, *p* = .266, *d* = 0.29, while we specifically expected a reduction for people with ASD for this latter category.

**Table 7:** Descriptive characteristics of the variables used in Study 5.

	Group	*N*	*M*	*SD*	*SE*
AQ**	ASD	33	2.24	0.35	0.06
	No ASD	29	2.00	0.31	0.06
Religiosity	ASD	33	2.31	1.75	0.31
	No ASD	29	2.22	1.45	0.27
Religious Behaviours	ASD	33	1.94	1.25	0.22
	No ASD	29	2.01	1.37	0.25
CREDs	ASD	33	2.42	1.42	0.25
	No ASD	29	2.10	1.13	0.21
Intentional	ASD	33	78.53	16.24	2.83
	No ASD	29	73.59	18.10	3.36
Random*	ASD	33	27.40	22.94	3.99
	No ASD	29	39.86	22.77	4.23
Mechanical*	ASD	33	15.53	18.23	3.17
	No ASD	29	31.09	24.71	4.59

* *p* < .05,

** *p* < .005, ASD = Autism Spectrum Disorder, No ASD = adolescents without Autism Spectrum Disorder, AQ = Autism Quotient, CREDs = Credibility Enhancing Displays Scale, Intentional, Random and Mechanical refer to the Geometrical Figures Task videos.

### Discussion

In Study 5, we observed that adolescents with ASD did not differ from adolescents without ASD on religiosity, religious behaviors, CREDs or intentional videos, but did differ on random and mechanical videos in the sense that they attributed less intentionality towards these latter videos. Following suggestions of Swanson [39] we hypothesized that religiosity in autistic people may perhaps be somewhat more oriented towards religious behavior (i.e., in the form of ritualized behaviors), but we found no support for this idea. With regards to the absence of a difference on the religiosity measures, our study deviates from the findings of Caldwell-Harris et al. [33] and Norenzayan et al. [16] who did observe differences between people with and without ASD. Our findings were comparable to those of Reddish et al. [36] who observed only very few differences between the people with and without ASD on seven measures of religious beliefs and behaviors. These findings indicate that at least in the Netherlands, mentalizing deficiencies were not associated with disbelief. Further, we observed that adolescents with ASD attributed less intentionality to mechanical and random videos. This is in line with the idea that people with autism are better in systemizing [24,55]. Interestingly, adolescents with ASD did not seem to have difficulties with attributing intentionality to intentional movements. However, as pointed out in the discussion of Study 4 as well, it is possible that participants with ASD conducted the task using a systematic strategy (i.e., maximizing the contrast in ratings between videos from different categories). Thus, for future studies, it may be practical to establish what type of intentionality people with ASD perceive in the videos in a more qualitative approach, instead of working with a scale from 1 to 100.

## General discussion

In four large sample studies from three countries and a small sample study involving people with ASD we found mixed evidence for a relationship between mentalizing and religiosity. Importantly, we could not replicate the finding that the AQ was predictive of religiosity in any of the studies. Only when fitting a curvilinear model we observed that high scores on the AQ were related to decreased levels of religious belief, but only in the US sample. In addition, correlations between religiosity and all other variables were higher in the US than in the other samples. Further, we directly compared different measures of mentalizing (i.e., the AQ, EQ, EQ-SQ and GFT) with demographical characteristics (i.e., gender, age and education) and cultural learning variables (i.e., CREDs). We found that mentalizing and hyper-systemizing only made a small contribution to predicting religious beliefs in the US, whereas gender, age and CREDs made robust contributions in the Netherlands and the US. Furthermore, in a Dutch sample we found no differences in terms of the strength of religious beliefs between people with and without strong mentalizing deficiencies. In short, the current studies highlight the importance of culture for determining religious beliefs in two respects. First, when explaining supernatural beliefs, the influence of cultural learning seems more important than individual characteristics such as gender, age and mentalizing. Second, even if mentalizing explains anything about supernatural beliefs, it could be that this is only the case in countries where believing is normative.

Overall, the current findings add to recent work in which authors questioned whether mentalizing was related to religious beliefs [25,26,36,85]. Although we did find a curvilinear effect, we only observed this for the AQ, not for any other of the operationalizations of mentalizing (i.e., the EQ, GFT or EQ-SQ). One intriguing possibility would be to analyze previously published data [16,25,26,33,36] using curvilinear models on the AQ, to see whether it this theoretical suggestion can be replicated in other samples as well. However, the findings that people with ASD did not differ from people without ASD [36] in a US sample makes a strong case against the idea that only strong mentalizing deficiencies are inversely related to supernatural beliefs in countries where religiosity is normative.

In Study 5, we conducted a similar study and we observed that when comparing adolescents with and without ASD, we did not find any difference in terms of the level of religiosity. These findings coincide with anecdotal reports that showed that people with ASD are in fact capable of believing in supernatural agents [8,35,37–39] and our findings are in accordance with the findings of other researchers investigating the level of religiosity in people with ASD [34–36]. Our observations contrast to the findings of two previous investigations involving the religiosity of people with ASD. However, it is important to note that these findings were based on a small sample (*N* = 12 vs. *N* = 13; [16]) or were obtained by comparing forums of websites [33], which may not have resulted in representative samples.

Interestingly, our findings on the GFT in Study 4 showed that people with ASD were as capable as people without ASD in attributing intentionality to geometrical figures, but did attribute less intentionality to random and mechanical videos. This is somewhat in line with the findings of Gray et al., [34] who observed that people with ASD were better able to interact with nonhuman animals and robots than humans. According to the authors reading the intentions of these agents does not require deconstruction of complex social behaviors. A similar suggestion could be made regarding the reading the intentions of supernatural agents; in some cases, these might even be easier to understand (e.g., the 10 commands Mozes received from God), than the intentions of human agents [34]. Nevertheless, ASD is a very heterogeneous disorder so that generalizations should be made with caution [30].

Further, we observed in Study 4 that people without ASD who attributed more intentionality towards random and mechanical moving geometrical figures endorsed stronger religious beliefs. We hypothesized that attributing intentionality towards random and mechanical moving figures would require the activation of the ToM-network. If this is the case, these findings are in support of the idea that over-attribution of mentalizing capacities may underlie supernatural beliefs (e.g., 16). Alternatively, the data are in line with the theoretical idea that ontological confusions may underlie supernatural beliefs [25,63]. That is to say, people seem to have confused the distinctive attributes of mental, physical, living and animate phenomena (i.e., applying mental states to non-animate phenomena).

At least four limitations of the present studies are worth mentioning. First, our study may exaggerate the distinction between mentalizing skills and CREDs. Specifically, children likely need mentalizing skills in order to understand and represent the beliefs of their parents. Thus, there may be a strong interaction between mentalizing skills and CREDs that we could not capture by means of our regression analyses. Children that reason in a more mechanistic fashion may have a more difficult time to present their beliefs [86]. It is hard to disentangle mentalizing and mechanistic cognition from CREDs as they are an inherent part of the process whereby CREDs are acquired. A possible way of disentangling these concepts better is by means of a longitudinal study in which researchers follow children with more dominant mentalistic or mechanistic cognition and investigate how CREDs interact with these types of cognition. Relatedly, it would be interesting to study children or twins to investigate the heritability component of CREDs. There may be a common genetic factor that underlies both sensitivity to learning and practicing CREDs, which could partly explain the heritability component of religiosity and the observed effect of CREDs on religiosity.

Second, a shortcoming of the studies is that it is unclear to what extent we truly captured the construct mentalizing or other related processes (e.g., empathy, social skills etc.). Future research should focus on better tools to capture individual differences in mentalizing capacities. The effects of mentalizing on religiosity seem minor in the way we operationalized it, but it may still be worthwhile to further explore the effects of mentalizing in future research. For example, schizophrenic hallucinations and delusions are often characterized by magical and religious phenomena. It has been argued that this may be the result of too dominant mentalistic cognition and hyper-mentalizing [86].”

Third, we used different religiosity questions for all of the countries. To keep the questions as consistent as possible we operationalized religiosity by means of the questions that were comparable over the three countries (i.e., the questions related to belief in God, praying and church visit). However, these three questions may not have necessarily been the best ways to address the question of interest: Does an evolved cognitive mechanism for inferring intentionality (i.e., mentalizing) underlie the capacity of inferring the intentionality of supernatural agents? It could be argued that our measures of religiosity encompassed both intrinsic and extrinsic indicators of religiosity, whereas primarily intrinsic measures of religiosity may be related to mentalizing. To address this potential problem, we added network analysis models in all of the studies (except for Study 5 considering the small sample) and incorporated all questions that were related to religious beliefs, so that we could investigate whether some religiosity questions were stronger related to mentalizing than others. Also, we could investigate which AQ items specifically would underlie this relationship. However, only in the US sample some weak correlations were observed between the religiosity items and the AQ and EQ. This again supports the idea that there were cultural differences between the US and the European countries and future studies may address why exactly the relationship between operationalizations of mentalizing and religiosity seems stronger in one country than the other.

Fourth, in this study we compared CREDs with demographical factors and cognitive biases and it could be argued that this comparison between proximal and ultimate factors [66] in determining religious beliefs actually answers different questions. Proximal factors such as CREDs answer the question why people believe in supernatural agents nowadays. It is obvious that how your parents raised you is a strong determinant of one's personal worldview and religious beliefs [12,52]. Distal factors (e.g., cognitive biases as often discussed in the cognitive science of religion literature) could explain how supernatural beliefs once came into existence. How is it that at so many places on earth people independently started to believe in supernatural agents [87]? CREDs can help answer how, once one member of a tribe had supernatural beliefs, these beliefs were able to go ‘viral’ (i.e., quickly spread from one member of a tribe to another). However, CREDs cannot answer how this specific member started believing in supernatural agents for the first time. In that sense, it may be an unfair comparison to compare factors that are important to belief nowadays with factors that are nowadays no longer important but may have originated thousands of years ago. Nevertheless, as outlined in the introduction, the influence of mentalizing is often discussed in the literature (the current paper included) as if it distinguishes believers from non-believers nowadays [16,17,33]. So ultimately, the current studies show that nowadays there is a strong importance of cultural learning in the form of CREDs, and that variations in mentalizing are unlikely to discriminate believers from non-believers.

## Supporting information

S1 Appendix Explorative analysis 2: Direct replication. (DOCX)

S2 Appendix Items of all questionnaires used in the Network analyses models. (DOCX)
