# Supplementary material for: Mentalizing skills do not differentiate believers from non-believers, but credibility enhancing displays do
Source: PLoS One. 2017 Aug 23;12(8):e0182764. doi: 10.1371/journal.pone.0182764 (PMC5568287; doi:10.1371/journal.pone.0182764)
Supplement: S1 Appendix — (DOCX) [file pone.0182764.s001.docx]

**Supplementary material**

**S1 Appendix**

**Explorative analysis 2: Direct replication of [1]**

To investigate whether our differences with the study of Norenzayan et al. [1] could be attributed to differences in the analyses, we conducted a similar analysis as reported in the original study (Experiment 3a and 3b). Therefore, a logistic regression was conducted instead of a hierarchical logistic regression. Belief in a personal God was predicted by the AQ and gender, while controlling for age, education, income, EQ and SQ. Similarly as in the study of Norenzayan et al. [1], visual inspection of the data showed that religiosity was bimodally distributed, Kolmogorov-Smirnov(787) = .09, *p* < .001. Therefore, religiosity was dichotomized with a median split into low believers (average score lower than 3.71, 61.6%) and high believers (average score of 3.71 or higher, 38.4%). Compared to a constant only model, the first model was statistically significant, indicating that the predictors reliably distinguished between atheists and theists, χ^2^(7) = 38.31, *p* < .001, Nagelkerke *R^2^* = .08. Gender (Wald = 9.43, *B* = 0.02, *p* = .002), age (Wald = 9.69, *B* = 0.02, *p* = .002) and income (Wald = 5.01, *B* < 0.01, *p* = .025) all made a significant contribution whereas AQ (Wald = 0.65, B = -0.29, *p* = .422), education (Wald = 0.95, *B* = 0.01, *p* = .329), EQ (Wald = 0.02, *B* = 0.03, *p* = .881) and SQ (Wald = 1.03, *B* = -0.18, *p* = .311) did not. These results show that neither the type of analysis (i.e., hierarchical logistic regression instead of logistic regression) nor the specific variables included in the model can account for the lack of a relationship between AQ and religiosity.

**References**

[1] Norenzayan A, Gervais WM, Trzesniewski KH. Mentalizing deficits constrain belief in a personal God. PloS one 2012;7(5):e36880.
