# Supplementary material for: Mentalizing skills do not differentiate believers from non-believers, but credibility enhancing displays do
Source: PLoS One. 2017 Aug 23;12(8):e0182764. doi: 10.1371/journal.pone.0182764 (PMC5568287; doi:10.1371/journal.pone.0182764)
Supplement: S2 Appendix — (DOCX) [file pone.0182764.s002.docx]

**Supplementary material**

**S2 Appendix: Items of all questionnaires used in the Network analyses models**

**The Autistic-Spectrum Quotient [2], French validation [3], Dutch validation [4]**

**Used in Study 1 – 4**

1. I prefer to do things with others rather than on my own.
2. I prefer to do things the same way over and over again.
3. If I try to imagine something, I find it very easy to create a picture in my mind.
4. I frequently get so strongly absorbed in one thing that I lose sight of other things.
5. I often notice small sounds when others do not.
6. I usually notice car number plates or similar  strings of information.
7. Other people frequently tell me that what I’ve  said is impolite, even though I think it is polite.
8. When I’m reading a story, I can easily imagine  what the characters might look like.
9. I am fascinated by dates.
10. In a social group, I can easily keep track of several different people’s conversations.
11. I find social situations easy.
12. I tend to notice details that others do not.
13. I would rather go to a library than a party.
14. I find making up stories easy.
15. I find myself drawn more strongly to people  than to things.
16. I tend to have very strong interests, which  I get upset about if I can’t pursue.
17. I enjoy social chit-chat.
18. When I talk, it isn’t always easy for others to  get a word in edgeways.
19. I am fascinated by numbers.
20. When I’m reading a story, I find it difficult to  work out the characters’ intentions.
21. I don’ t particularly enjoy reading fiction.
22. I find it hard to make new friends.
23. I notice patterns in things all the time.
24. I would rather go to the theatre than a museum.
25. It does not upset me if my daily routine  is distubed.
26. I frequently find that I don’t know how to keep  a conversation going.
27. I find it easy to “read between the lines” when someone is talking to me.
28. I usually concentrate more on the whole picture, rather than the small details.
29. I am not very good at remembering phone numbers.
30. I don’t usually notice small changes in a situation,  or a person’s appearance.
31. I know how to tell if someone listening to me  is getting bored.
32. I find it easy to do more than one thing at once.
33. When I talk on the phone, I’m not sure when it’s  my turn to speak.
34. I enjoy doing things spontaneously.
35. I am often the last to understand the point of a joke.
36. I find it easy to work out what someone is  thinking or feeling just by looking at their face.
37. If there is an interruption, I can switch back to  what I was doing very quickly.
38. I am good at social chit-chat.
39. People often tell me that I keep going on and  on about the same thing.
40. When I was young, I used to enjoy playing games  involving pretending with other children.
41. I like to collect information about categories of things (e.g. types of car, types of bird,  types of train, types of plant, etc.).
42. I find it difficult to imagine what it would be  like to be someone else.
43. I like to plan any activities I participate in carefully.
44. I enjoy social occasions.
45. I find it difficult to work out people’s intentions.
46. New situations make me anxious.
47. I enjoy meeting new people.
48. I am a good diplomat.
49. I am not very good at remembering  people’s date of birth.
50. I find it very easy to play games with  children that involve pretending.

**Religiosity items derived from [16]**

1. I believe in God
2. When I am in troubleI find myself wanting to ask God for help
3. When people pray they are only talking to themselves (Reversed-coded)
4. I don’t really spend much time thinking about my religious beliefs (Reversed-coded)
5. I consider myself a spiritual believer (not a religious believer)
6. I often pray
7. I often visit a religious/spiritual institution (e.g. church)

**Self-constructed credibility enhancing displays items**

**Used in the Dutch sample, translated from Dutch (Study 2)**

1. To what extent did your parents/caretakers have a religious lifestyle during you upbringing?
2. To what extent did your parents/caretakers visit the church/musk or other religious institution?
3. To what extent was religiosity an affair of the family?
4. To what extent did your parents pray during dinner?
5. To what extent was Sunday seen as a sacred day of rest?
6. To what extent where religious ornaments/decorations part of the interior?
7. To what extent did your parents/caretakers wear religious clothing?

**Religiosity items used in the French sample (translated from French)**

**Used in the Swiss sample (Study 3)**

1. How do you define yourself religiously?

Please choose from: Christian, Jew, Muslim, Buddhist, Hindu, Atheist (not believer), Agnostic (we cannot know), Other

More than 2 times a month

1-2 times a month

Rarely or never

1. How often do you go to practice your religion in your cult place?
2. How often do you address your divinity or pray?

1 = not (important) at all, 7 = very important/ much

1. How important is religion in your daily life?
2. How important is it for you to belong to a religious community?
3. Is it easy to represent yourself God or/and his Will?
4. How much do you feel protected by God?
5. How much do you feel in relation with God?
6. Is religion a moral guide for you?
7. Do you believe in a form of spiritual existence after death?
8. How much does religion help you to know yourself better?
9. To what extent do you think your actions can be judged by a superior entity and there can be consequences for your life or afterlife?
10. To what extent is it important to you to generally practice your religiosity.

**Credibility enhancing displays scale [5]**

**Used in the US sample (Study 4)**

1. To what extent did your caregiver(s) attend religious services or meetings?
2. To what extent did your caregiver(s) engage in religious volunteer or charity work?
3. Overall, to what extent did your caregiver(s) act as good religious role models?
4. To what extent did your caregiver(s) make personal sacrificies to religion?
5. To what extent did your caregiver(s) act fairly to others because their religion taught them so?
6. To what extent did your caregiver(s) live a religiously ‘pure’ life?
7. To what extent did your caregiver(s) avoid harming others because their religion taught them so?

**References**

[1] Norenzayan A, Gervais WM, Trzesniewski KH. Mentalizing deficits constrain belief in a personal God. PloS one 2012;7(5):e36880.

[2] Baron-Cohen S, Wheelwright S, Skinner R, Martin J, Clubley E. The autism-spectrum quotient (AQ): Evidence from asperger syndrome/high-functioning autism, malesand females, scientists and mathematicians. J Autism Dev Disord 2001;31(1):5-17.

[3] Sierro G, Rossier J, Mohr C. Validation of the French Autism Spectrum Quotient scale and its relationships with schizotypy and Eysenckian personality traits. Compr Psychiatry 2016;68:147-155.

[4] Hoekstra RA, Bartels M, Cath DC, Boomsma DI. Factor structure, reliability and criterion validity of the Autism-Spectrum Quotient (AQ): a study in Dutch population and patient groups. J Autism Dev Disord 2008;38(8):1555-1566.

[5] Lanman JA, Buhrmester MD. Religious actions speak louder than words: exposure to credibility-enhancing displays predicts theism. Religion, Brain & Behavior 2015:1-14.
